# Stage IVB ovarian carcinosarcoma in BRCA wild-type patients: two case reports of unexpected long-term remission

**DOI:** 10.3389/fonc.2025.1728398

**Published:** 2026-01-09

**Authors:** Orazio De Tommasi, Sofia Bigardi, Angela Guerriero, Giulia Tasca, Davide Massa, Giulia Spagnol, Margherita Nardin, Marco Noventa, Carlo Saccardi, Roberto Tozzi

**Affiliations:** 1 Unit of Gynecology and Obstetrics, Department of Women and Children’s Health, University of Padua, Padua, Italy; 2 Surgical Pathology Unit, University Hospital of Padova, Padova, Italy; 3 Division of Oncology 2, Istituto Oncologico Veneto Istituto di Ricovero e Cura a Carattere Scientifico (IRCCS), Padova, Italy; 4 Radiology Unit, Istituto Oncologico Veneto Istituto di Ricovero e Cura a Carattere Scientifico (IRCCS), Padova, Italy

**Keywords:** niraparib, ovarian cancer, ovarian carcinosarcoma, primary debulking surgery, tamoxifen

## Abstract

**Background:**

Ovarian carcinosarcoma (OCS), also known as malignant mixed Müllerian tumor, is a rare and highly aggressive subtype of epithelial ovarian cancer, accounting for less than 4% of all cases. It typically presents at advanced stages and is associated with dismal outcomes, with a five-year survival rate below 30%. Despite improvements in cytoreductive surgery and systemic therapies, long-term survival in stage IV disease remains exceedingly uncommon.

**Case presentation:**

We report two exceptional cases of stage IVB Müllerian carcinosarcoma occurring in BRCA wild-type postmenopausal women who achieved prolonged complete remission exceeding five years after multimodal management. The first patient, aged 61, presented with bilateral adnexal masses and a solitary pulmonary metastasis. She underwent primary cytoreductive surgery including hysterectomy, bilateral adnexectomy, lymphadenectomy, appendicectomy, and omentectomy, followed by six cycles of platinum-taxane chemotherapy. Residual pulmonary disease was later removed via video-assisted thoracoscopic lobectomy, confirming metastatic OCS. Post-recurrence, she received off-label maintenance with tamoxifen 20 mg daily for five years and remains disease-free at 70 months. The second patient, aged 70, presented with a pelvic mass invading the recto-sigmoid wall and a synchronous hepatic metastasis. She underwent extensive cytoreductive surgery including hysterectomy, en-bloc rectal resection, lymphadenectomy, cholecystectomy, and liver wedge resection, achieving complete macroscopic cytoreduction. Histology confirmed a Müllerian carcinosarcoma with a predominant endometrioid component. Postoperative chemotherapy with carboplatin-paclitaxel was followed by maintenance niraparib 100 mg twice daily for three years. She remains in complete remission at 60 months.

**Discussion:**

Both patients demonstrate durable disease control in the absence of germline or somatic BRCA mutations, suggesting that long-term remission may be achievable even in BRCA-wild-type OCS through optimal surgery and individualized maintenance approaches. Tamoxifen, rarely employed in this setting, may have provided estrogen-receptor-mediated tumor suppression in the first case, while the second case highlights potential activity of PARP inhibition beyond BRCA mutation carriers.

**Conclusion:**

These two reports challenge the long-held perception of uniformly poor outcomes in metastatic ovarian carcinosarcoma. Complete cytoreductive surgery combined with tailored systemic and maintenance therapies can achieve sustained remission even in advanced-stage BRCA-wild-type patients. Broader molecular profiling and international collaboration are essential to refine management strategies for this rare and aggressive malignancy.

## Introduction

Ovarian cancer remains the deadliest gynecologic malignancy, ranking fifth among cancer-related deaths in women worldwide. Despite advances in cytoreductive surgery and systemic therapy, the five-year survival rate for advanced-stage epithelial ovarian cancer (EOC) remains below 30% (1–3). Among these, ovarian carcinosarcoma (OCS), also known as malignant mixed Müllerian tumor, is an exceptionally rare and highly aggressive histologic subtype, accounting for approximately 2% of all EOCs (4). Defined by the biphasic presence of both high-grade carcinomatous and sarcomatous elements, OCS demonstrates distinct biological behavior and clinical outcomes compared to other ovarian cancers. Molecular data support a monoclonal epithelial origin for both components, classifying OCS as a variant of high-grade epithelial neoplasia rather than a true mixed tumor (5).

OCS typically presents in postmenopausal women with bulky disease and is diagnosed at an advanced stage (FIGO III–IV) in over 70% cases (6).

In this report, we present two cases of Müllerian carcinosarcoma with visceral metastases (stage IVB) at diagnosis, both of whom achieved long-term complete remission exceeding five years following aggressive multimodal treatment. Each case underscores a different therapeutic strategy: one using extended hormonal maintenance with tamoxifen, and the other receiving long-term PARP inhibition with niraparib. These observations challenge historical assumptions about the uniformly poor prognosis of OCS and raise important considerations regarding individualized treatment approaches, surgical aggressiveness, and the evolving role of maintenance therapy.

## Case 1

**Figure 1:**
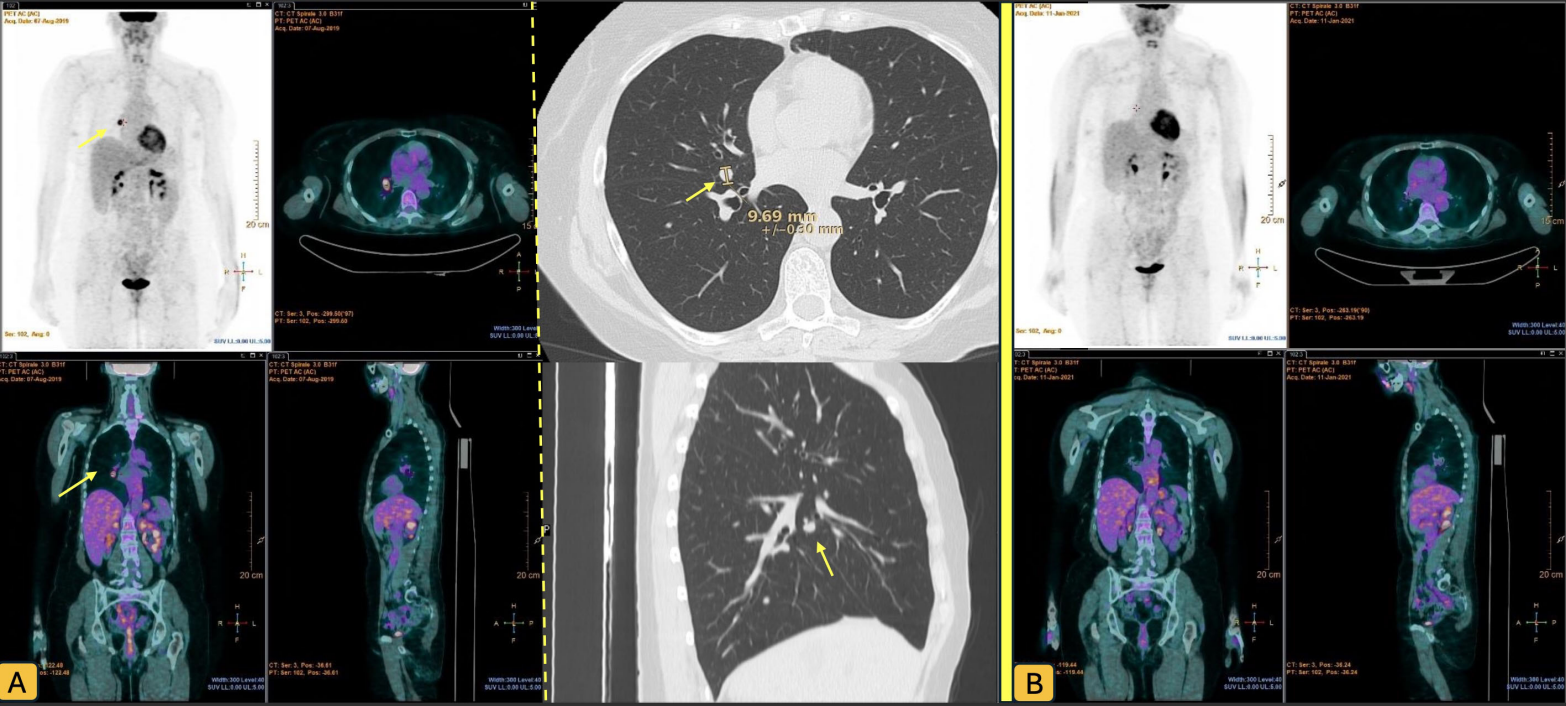
PET CT scan of patient 1 before **(A)** and after **(B)** lung lobectomy. Yellow arrows show the Pulmunary nodule.

A 61-year-old female presented to our center in December 2016 following the incidental ultrasound detection of a 7 cm multilobulated mass involving the left adnexa and a 5 cm mass on the right. Preoperative CT also revealed a 12 mm right middle lobe hilar pulmonary nodule (**Figure 1**). She underwent bilateral adnexectomy with intraoperative frozen section, which revealed a lesion highly suspicious for malignancy. Based on this finding, comprehensive surgical staging was performed, including total hysterectomy, bilateral adnexectomy, pelvic lymphadenectomy, appendicectomy, and infracolic omentectomy. Intraoperatively, a 3 cm omental nodule on the sigmoid colon was also resected.

Final histopathological examination diagnosed in the left annex an OCS (WHO 2020 classification), composed predominantly (>90%) of high-grade serous carcinoma with cribriform and solid nested growth patterns, and a minor component of high-grade homologous sarcoma. Both epithelial and mesenchymal elements demonstrated marked cytologic atypia, including nuclear gigantism, atypical mitoses, and extensive necrosis. The tumor infiltrated the peritoneum and peri-adnexal soft tissues. Tumor cells of the carcinomatous component showed positive immunostaining for WT1, for EMA and for estrogen receptor (80%) and progesterone receptor (50%); sarcomatous component showed negative immunostaining for epithelial markers and for mesenchymal markers. P53 showed immunostaining positivity in a mutated-pattern in both components (**Supplementary Table 1**). In the right annex an high-grade serous carcinoma was diagnosed; with diffuse lymph vascular invasion and extension to peri-ovarian soft tissues. Peritoneal samples demonstrated metastasis of carcinosarcoma. Molecular analysis revealed microsatellite stability (MSS) and non-informative BRCA testing.

The patient underwent six cycles of adjuvant chemotherapy with cisplatin and paclitaxel, resulting in a partial response of the hilar pulmonary lesion. Interval imaging over the following months demonstrated slow regrowth of the nodule. PET/CT confirmed isolated hypermetabolic activity in the right hilum without evidence of other disease. She was therefore scheduled for video-assisted thoracoscopic lobectomy of the right middle lobe.

The lesion was visualized posterior to a venous branch of the middle lobe, necessitating lobectomy for complete excision. An ilomediastinal lymphadenectomy was performed, sparing station 4R due to absence of visible lymphadenopathy.

Histology confirmed a pulmonary metastasis from tubo-ovarian carcinosarcoma, with solid/organoid architecture, 70% necrosis, high mitotic index (60/2 mm²), and both vascular and perineural invasion. Immunohistochemistry was consistent with Müllerian origin, with positivity for WT1, PAX8, MNF116, 34βE12, and estrogen receptor (80%). All examined lymph nodes were negative for metastasis.

No chemotherapy was administered post-recurrence. Instead, the patient began an off-label hormonal maintenance regimen with tamoxifen 20 mg daily, which she continued for five years. Clinical follow-up included quarterly CA-125 monitoring for the first three years, followed by semi-annual assessments for two years. Imaging consisted of annual PET/CT alternating with thoracoabdominal CT. At the end of the five-year post-recurrence follow-up, the patient remained disease-free and transitioned to annual surveillance with CA-125 and thoracoabdominal imaging.

At last evaluation, she had achieved a progression-free survival (PFS) of 70 months (**Table 1**).

**Table 1:** Clinical, pathological, and treatment characteristics of the two reported cases of stage IVB ovarian carcinosarcoma.

Feature	Case 1	Case 2
Stage	IVB	IVB
Age	61	79
Progression-Free Survival (PFS)	70 months	60 months
Maintenance Therapy	Tamoxifen 20 mg/day for 5 years (off-label)	Niraparib for 3 years
Chemotherapy	6 cycles carboplatin (AUC5) + paclitaxel	6 cycles carboplatin (AUC5) + paclitaxel
Histology	Tubo-ovarian carcinosarcoma, >90% high-grade serous carcinoma, high-grade homologous sarcoma	Müllerian carcinosarcoma, >90% poorly differentiated endometrioid carcinoma, high-grade homologous sarcoma (<10%)

## Case 2

**Figure 2:**
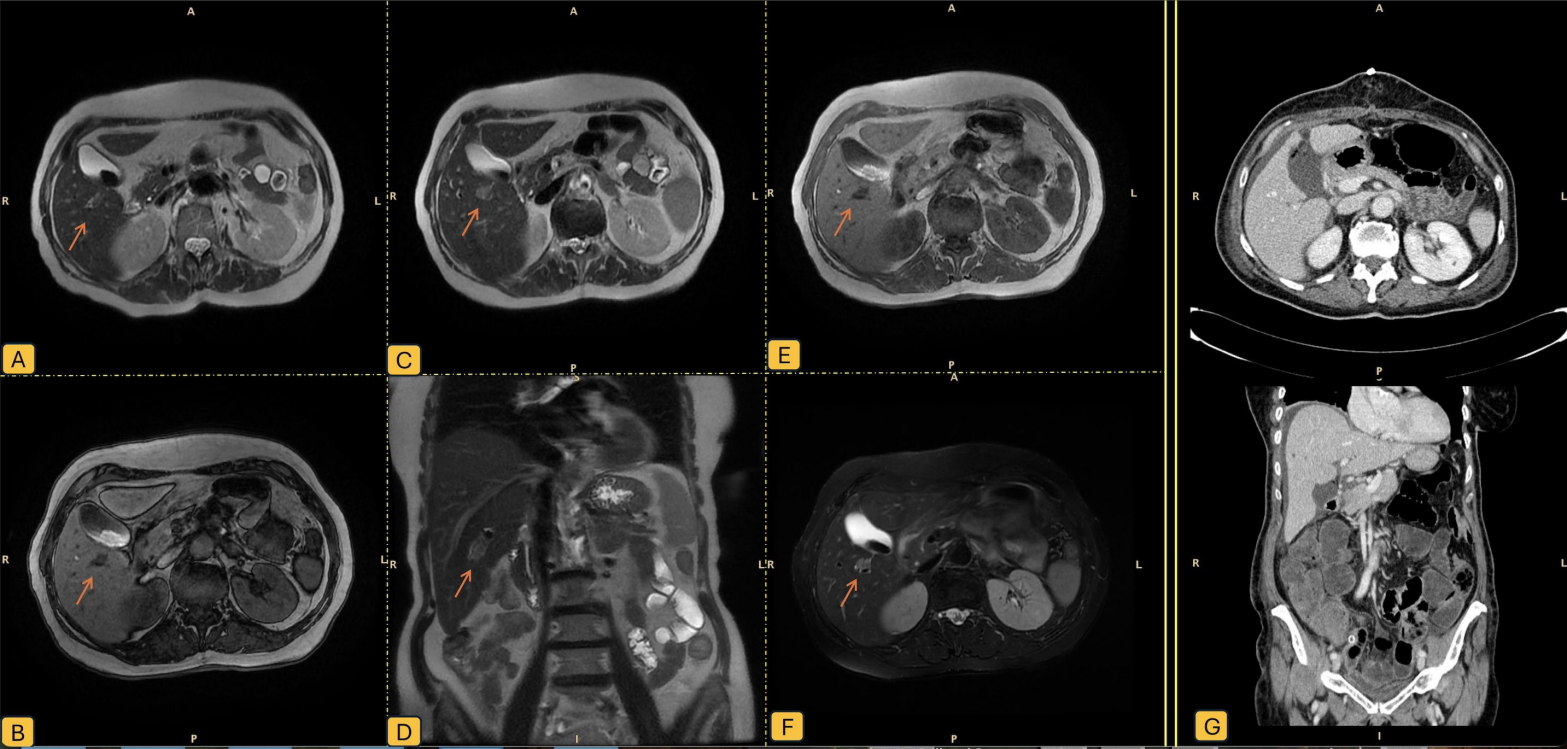
Liver lesion at diagnostic imaging; **(A)** T2-weighted images using single shot fast spin echo sequence (SSFE); **(B)** T1-weighted sequence; **(C)** another T2 SSFSE sequence ; **(D)** coronal T2 SSFE; **(E)** T1-weighted gradient-echo in-phase; **(F)** T2 Fast Spin Echo with Fat Saturation (FRFSE FATSAT); **(G)** TC scan after cytoreductive surgery. Orange arrow shows the epatic nodule.

A 70 year-old female patient presented in February 2020 with a large pelvic mass (14 × 9 cm) centered in the uterus and cervix. Imaging showed a recto-sigmoid wall invasion and a 22 mm lesion in segment V of the liver with radiological features consistent with metastasis (**Figure 2**). MRI confirmed infiltration of the rectum and sigma. CA-125 was within normal range (10.1 kU/L), and liver needle-biopsy revealed a poorly differentiated solid carcinoma compatible with gynecologic origin.

She underwent extensive cytoreductive surgery via midline laparotomy. Intraoperatively, a friable tumor mass was identified infiltrating the posterior uterine wall, upper posterior vaginal wall, and mesorectum. Left annex and right ovary were grossly normal, right fallopian tube was not identifiable. The pelvic mass was tightly adherent to the rectum and posterior vaginal wall, requiring en-bloc resection. Mobilization of the sigmoid colon and ligation of the inferior mesenteric artery were performed. Rectal resection was achieved using a mechanical stapler. Vaginal dissection and careful separation of the rectovaginal septum allowed for isolation and removal of the entire mass, uterus, adnexa, and rectum en-bloc. An enlarged intercavo-aortic lymph node was excised. Intraoperative liver ultrasound identified a pericholecystic hypoechoic nodule. Due to concomitant cholelithiasis, cholecystectomy was performed, and the liver lesion was removed through a wedge resection. The diaphragm, paracolic gutters, and upper abdomen were free of disease.

Histopathology examination of the pelvic mass revealed a carcinosarcoma of the right fallopian tube with a predominantly (>90%) poorly differentiated endometrioid-type carcinoma and a minor (<10%) component of high-grade homologous sarcoma (**Figure 3**). Extensive necrosis and multifocal vascular invasion were observed, with infiltration of the outer third of the myometrium and rectal wall up to the submucosa. Immunohistochemical analysis demonstrated diffuse positive staining of the endometrioid component for Cytokeratin MNF116, Cytokeratin AE1/AE3, Cytokeratin 7, EMA, PAX8 and vimentin, patchy positivity for p16; cells were negative for WT1, Cytokeratin 20, CDX2, TTF1, and S100. P53 showed immunostaining positivity in a mutated-pattern in both components (**Supplementary Table 1**). Liver excision confirmed metastatic involvement (Stage FIGO IVB). Molecular testing showed MSS status and non-informative and non-informative sBRCA.

**FFigure 3:**
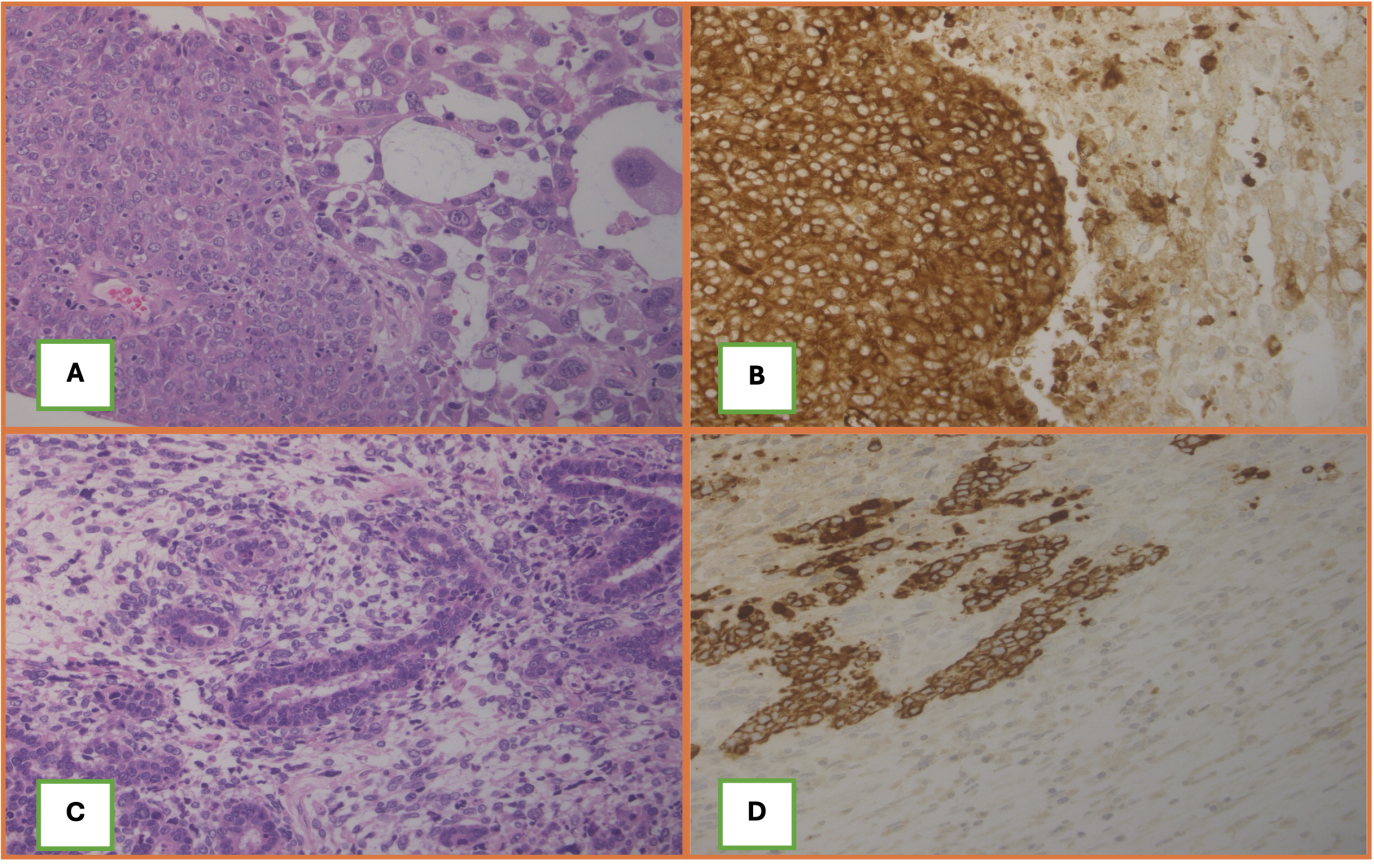
**(A, C)** Histological aspect of both tumors with a biphasic morphology with carcinomatous and sarcomatous components. **(B, D)** Cytokeratin AE1/AE3 is diffusely expressed in the carcinomatous component and patchy in the sarcomatous part.

The patient completed six cycles of carboplatin (AUC5) and paclitaxel (175 mg/m²) every 21 days. Post-treatment CT non evidence of disease. CA-125 remained stable at 12 kU/L.

She subsequently commenced maintenance therapy with niraparib 100 mg bid as per standard therapeutic use, continued for three years without significant toxicity. At last follow-up, the patient remained disease-free, with a PFS of 60 months (**Table 1**).

## Discussion

OCS, also known as malignant mixed Müllerian tumor, is a rare and highly aggressive malignancy representing approximately 1%–4% of epithelial ovarian cancers. Characterized by the presence of both high-grade epithelial (carcinomatous) and mesenchymal (sarcomatous) components, OCS has traditionally been associated with poor clinical outcomes and limited therapeutic options. A synthesis of the current literature (including retrospective cohorts, registry-based analyses, and molecular case series) reveals significant heterogeneity in survival outcomes and a shifting therapeutic landscape influenced by molecularly targeted treatments.

Historically, patients with OCS exhibited a dismal prognosis, with median overall survival (OS) ranging from 8.2 to 12.7 months in cohorts lacking molecular stratification and maintenance therapy options (6, 7). These earlier studies reported no cases of long-term survival among stage IV patients, and progression was nearly universal within one year (8). However, more recent data suggest a marked improvement in outcomes, particularly in molecularly selected populations. Liang et al. (9) documented a median OS of 40 months in a cohort of 51 patients, including three patients with stage IVB disease who achieved durable complete remission exceeding 30 months following surgery, platinum-based chemotherapy, and maintenance therapy with the PARP inhibitor olaparib. Similar findings were described by Nienhaus et al. (10) who reported a patient with stage IVB OCS and germline BRCA1 mutation achieving a complete response and remaining disease-free beyond three years on olaparib maintenance. Zheng et al. (11) likewise reported long-term survival (≥30 months) in nine patients, though molecular status was not uniformly described.

Data from the Korean national cancer registry (12) further support the existence of long-term survivors in advanced-stage disease. Among 237 patients with distant-stage OCS, the five-year OS was 32.8%, though the study lacked molecular and treatment-specific annotations. Still, these registry findings underscore that durable survival is achievable even in metastatic settings, likely linked to improvements in surgical techniques, chemotherapy regimens, and possibly molecularly guided interventions.

The presented cases highlight the potential for prolonged disease control in metastatic tubo-ovarian carcinosarcoma through multimodal treatment, including complete abdominal cytoreductive surgery and metastasectomy via thoracoscopy in the first case.

Regarding maintenance therapy, olaparib is the only agent consistently associated with durable complete responses in BRCA-mutated OCS patients. No such benefit has been documented for niraparib, which failed to provide long-term disease control in the only reported case (9). Tamoxifen has not been employed in any published OCS case series or trials to date. These findings align with current NCCN guidelines (13), which recommend BRCA1/2 testing in all high-grade epithelial ovarian cancers, including OCS, and the use of PARP inhibitors as maintenance therapy in mutation-positive cases. Case studies have shown concordant BRCA2 mutations in both epithelial and sarcomatous tumor components (14) supporting a monoclonal origin and the theoretical responsiveness of both compartments to PARP inhibition.

## Patient perspective

In conclusion, while OCS remains an aggressive and often chemoresistant tumor with overall inferior survival compared to serous ovarian carcinoma, recent evidence demonstrates that long-term complete remission is possible in select patients with advanced-stage disease, particularly those harboring BRCA mutations and receiving olaparib maintenance following platinum-based chemotherapy. Complete surgical resection of all visible disease, combined with the routine integration of molecular profiling and maintenance therapy, appears to be redefining the prognosis of this historically lethal malignancy. However, the rarity of the disease continues to limit prospective trial data, emphasizing the urgent need for international collaboration and inclusion of OCS in biomarker-driven treatment studies.

## Data Availability

The original contributions presented in the study are included in the article/**Supplementary Material**. Further inquiries can be directed to the corresponding author.

## Supplementary material

Supplementary Table 1 Immunohistochemistry Panel. Yellow: Positive: Blue: negative: Black: not performed.
