# Supplementary material for: Stage IVB ovarian carcinosarcoma in BRCA wild-type patients: two case reports of unexpected long-term remission
Source: Front Oncol. 2026 Jan 9;15:1728398. doi: 10.3389/fonc.2025.1728398 (PMC12827093; doi:10.3389/fonc.2025.1728398)
Supplement: Supplementary Table 1 — Immunohistochemistry Panel. Yellow: Positive: Blue: negative: Black: not performed. [file Table1.docx]

**Supplementary Table 1 (S1)**

| **Marker** | **Case 1 (Tubo-ovarian carcinosarcoma)** | **Case 2 (Müllerian carcinosarcoma)** |
| --- | --- | --- |
| WT1 |  |  |
| p53 |  |  |
| Estrogen receptor (ER) |  |  |
| Progesterone receptor (PR) |  |  |
| CD10 |  |  |
| Vimentin |  |  |
| Epithelial membrane antigen (EMA) |  |  |
| MNF116 |  |  |
| PAX8 |  |  |
| AE1/AE3 | Not specified |  |
| CK7 | Not specified |  |
| p16 | Not specified |  |
| Desmin |  |  |
| MYF4 |  |  |
| HHF35 |  |  |
| CK20 | Not specified |  |
| CDX2 |  |  |
| TTF1 |  |  |
| p40 |  | Not specified |
| MIB1 (Ki-67) |  | Not specified |
| CD56 |  | Not specified |
| Calretinin | Not specified |  |
| HBME-1 | Not specified |  |
| GATA3 | Not specified |  |
| GCDFP15 | Not specified |  |
| Chromogranin |  |  |
| Synaptophysin |  |  |
| HMB45 |  |  |
| MelanA |  |  |
| Actin muscle-specific (ActML) |  |  |
| CEA | Not specified |  |
| CD117 | Not specified |  |
| PLAP | Not specified |  |
| Alpha-fetoprotein | Not specified |  |

Table S1: Immunohistochemistry Panel. Yellow: Positive: Blue: negative: Black: not performed
